# Total hip arthroplasty for Crowe IV hip without subtrochanteric shortening osteotomy -a long term follow up study

**DOI:** 10.1186/1471-2474-15-72

**Published:** 2014-03-10

**Authors:** Toshiyuki Kawai, Chiaki Tanaka, Hiroshi Kanoe

**Affiliations:** 1 Department of Orthopaedic Surgery, Kyoto City Hospital, 1-2, Higashitakada-cho, Mibu, Nakagyo-ku, Kyoto 604-8845, Japan; 2 Department of Orthopedic Surgery, Kyoto University Graduate School of Medicine, 54 Shogoin-Kawaharacho, Sakyo-ku, Kyoto 606-8507, Japan

## Abstract

**Background:**

Several authors reported encouraging results of total hip arthroplasty (THA) for Crowe IV hips performed using shortening osteotomy. However, few papers have documanted the results of THA for Crowe IV hips without shortening osteotomy. The aim of the present study was to assess the long term-results of cemented THAs for Crowe group IV hips performed without subtrochanteric shortening osteotomy.

**Methods:**

We have assessed the long term results of 27 cemented total hip arthroplasty (THA) performed without subtrochanteric osteotomy for Crowe group IV hip. All THAs were performed via transtrochanteric approach.

**Results:**

After a mean follow-up of 10.6 (6 to 17.9) years, 25 hips (92.6%) had survived without revision surgery and survivorship analysis gave a survival rate of 96.3% at 10 years with any revision surgery as the end point. Although mean limb lengthening was 3.2 (1.0 to 5.1) cm, no hips developed nerve palsy. Complications occurred in four hips, necessitating revision surgery in two. Among the four complications, three involved the greater trochanter, two of which occurred in cases where braided cables had been used to reattach the greater trochanter.

**Conclusions:**

Although we encountered four complications, including three trochanteric problems, our findings suggest that THA without subtrochanteric shortening osteotomy can provide satisfactory long-term results in patients with Crowe IV hip.

## Background

Total hip arthroplasty (THA) is an effective operation to relieve pain and improve function in an osteoarthritic hip [1]. Charnley suggested that untreated congenital dislocation of the hip is a contraindication for THA because of a lack of acetabular bone stock [2], raising concern about the higher rate of aseptic loosening of the acetabular component in subluxed hips.

Placement of the acetabular component in the true acetabulum has yielded the most durable results of THA in patients with developmental dysplasia of the hip [3-6]. In Crowe group IV hips, however, anatomic socket placement can make hip reduction difficult, and reduction may cause considerable limb lengthening and increase the risk of neurologic traction injury [2,7]. Therefore, femoral shortening is utilized in some cases to facilitate reduction, and protect the sciatic nerve.

Several authors reported encouraging results of THA for Crowe IV hips performed using shortening osteotomy [8-10]. However, to the best of our knowledge, few papers have documented the results of THA for Crowe IV hips without shortening osteotomy [11,12]. The aim of the present study was to retrospectively assess the long term-results of cemented THAs for Crowe group IV hips performed without subtrochanteric shortening osteotomy.

## Methods

Between August 1992 and November 2004, we performed 38 THAs in 32 patients for Crowe group IV developmental hip dysplasia. Among them, 6 hips (15.8%) in 5 patients required subtrochanteric shortening osteotomy and were excluded from the study. Shortening osteotomy was performed only when reduction into the true acetabulum seemed otherwise impossible. These six hips were excluded so that the results would be free from any influence of shortening osteotomy. Among the remaining 32 hips, 5 were excluded because of short follow-up period of less than 6 years: the 3 patients concerned (5 hips) had died of diseases unrelated to their hip problems. This left 27 hips in 24 patients for analysis.

All the procedures were carried out by the senior author (C.T.). The mean age of the patients at the time of the index THA was 57.6 (44 to 80) years. For 23 hips, THA was the first procedure; the remaining 4 hips in 4 patients had previously been treated surgically (femoral osteotomy) before the index THA.

In 16 hips, a CMK (Biomet, Warsaw, IN) cup was used. In the remaining hips, 7 PHS (Kyocera, Kyoto, Japan) cups and 4 small Charnley cups (Thackery, Leeds, UK) were used. The femoral device used was a CMK in 14 hips, and a PHS 6 in 13 hips. A 22 mm femoral head was always used. Both components were cemented with CMW (DePuy, UK).

The clinical assessments were performed using the Japanese Orthopedic Association (JOA) hip score, which allocates 40 points for pain, 20 points for range of movement, 20 points for walking ability, and 20 points for activities of daily living, with a maximum total score of 100 points. The patients were also examined for the presence of Trendelenburg sign preoperatively and at final follow up.

Radiographic analysis was performed on serial anteroposterior radiographs of the pelvis. On the pelvic side, the presence and evolution of radiolucent lines according to DeLee and Charnley [13] were noted. On the femoral side, investigated parameters included the evolution of radiolucent lines in the 7 zones of the femur [14]. Loosening was defined according to the criteria of Johnston et al. [15] as definite, probable, or possible.

**Figure 1:**
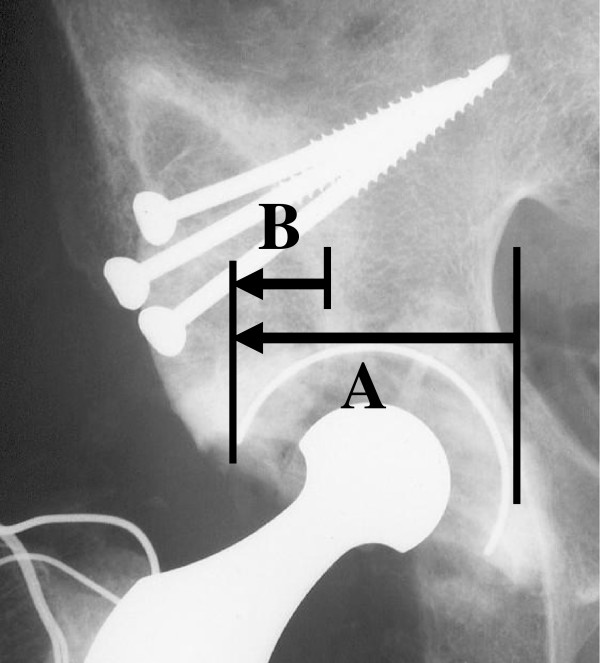
Measurement of percentage of bone graft for the acetabulum, calculated as the ratio of B to A.

The percentage of structural bone graft for the acetabulum was calculated as the ratio of horizontal length of the graft covering the cup to the horizontal length of the cup (Figure 1). Consolidation and incorporation of the graft was assessed using the criteria of Conn et al. [16] and was defined as identical density of the graft and host bone with a continuous trabecular pattern throughout the graft-host bone junction.

We referenced the location of the femoral head center preoperatively and postoperatively to a line drawn through the teardrops. The vertical height of the femoral head center perpendicular to this reference line was measured. The horizontal distance between the lowest point of the teardrop and femoral head center was measured.

Limb lengthening was defined as the brought down distance of the prominent point of the lesser trochanter that was obtained by comparing the postoperative and preoperative radiographs and corrected according to the magnification. Leg length discrepancy was assessed preoperatively and at final follow up with reference to the lesser trochanter.

The study was conducted according to the Declaration of Helsinki [17]. The study protocol was approved by the Ethical Review Board, Kyoto City Hospital, Kyoto, Japan (reference number 188).

### Surgical procedure

THA was carried out in the lateral decubitus position, via the transtrochanteric approach. The joint capsule, fibrous scar tissue, shelf, and osteophytes were removed carefully and completely. The primary landmark to identify was the inferior margin of the acetabulum. After excision of fibrous tissue, the pulvinar, which usually covers the inferior margin of the acetabulum, was recognized as a landmark in all cases. The acetabulum was prepared to create a hemispherical bone cavity with curved gouges and then reamed carefully. A bone autograft obtained from the femoral head and neck was used to augment and reinforce the roof of the undeveloped original acetabulum in all of the 27 procedures. The graft was fixed with at least two screws on the superolateral acetabular defect so that it could cover especially the posterior superior portion of the defect. A socket with an outer diameter of 37 to 40 mm was cemented into the acetabular cavity.

The trial femoral component was then inserted with the hip dislocated anteriorly and the lower leg aligned vertically, and then the anteversion of the stem was determined in this positioning. After the trial components had been placed, trial reduction was attempted. In high dislocations, soft tissue or neurovascular tension sometimes prevents reduction of the femoral head. When a trial reduction was impossible, the additional femoral neck cut was performed until femoral head reduction was possible. Reduction was performed with the hip and the knee both flexed at approximately 30 degrees. It was not achieved by pulling the leg distally but by using a head pusher (Biomet, Warsaw, IN) to push the inserted stem head distally. After reduction, the sciatic nerve tension was assessed by palpation with the knee flexed at approximately 30 degrees. Although reduction was usually tight, muscle release or tenotomy was not performed.

The greater trochanter was reattached with three or four monofilament stainless steel wires in all the hips except three hips; in two hips cable grip and Dall-Miles braided cables were used and in one hip monofilament titanium wires were used instead. The wiring technique consists of three vertical wires and one transverse wire [18]. Patients were free to walk with two supports after two days. Full weight-bearing was usually allowed after six weeks.

### Statistical analysis

Paired t-test was used to assess the change in JOA score. Additionally, considering relatively small sample size, *post-hoc* power analysis was performed to examine the statistical power for the comparison between preoperative and postoperative JOA scores. For statistical analysis, JMP 9 software (SAS Institute, Cary, NC, USA) was used. Differences at *p* < 0.05 were considered statistically significant. The endpoint for survival was defined as any revision surgery. Survival was determined using the actuarial life-table constructs described by Kaplan and Meier.

## Results

The mean follow-up period was 10.6 (6.0-17.9) years. The JOA hip score was increased from a mean value of 41.8 (17–65) preoperatively to 80.0 (59–96) at the last follow-up and this difference was statistically significant (*p* < 0.001). The JOA score improved in all hip. The statistical power for the analysis of difference between preoperative and postoperative JOA score was more than 0.99.

The Trendelenburg sign was positive in all hips preoperatively and five hips (19%) at final follow up.

**Figure 2:**
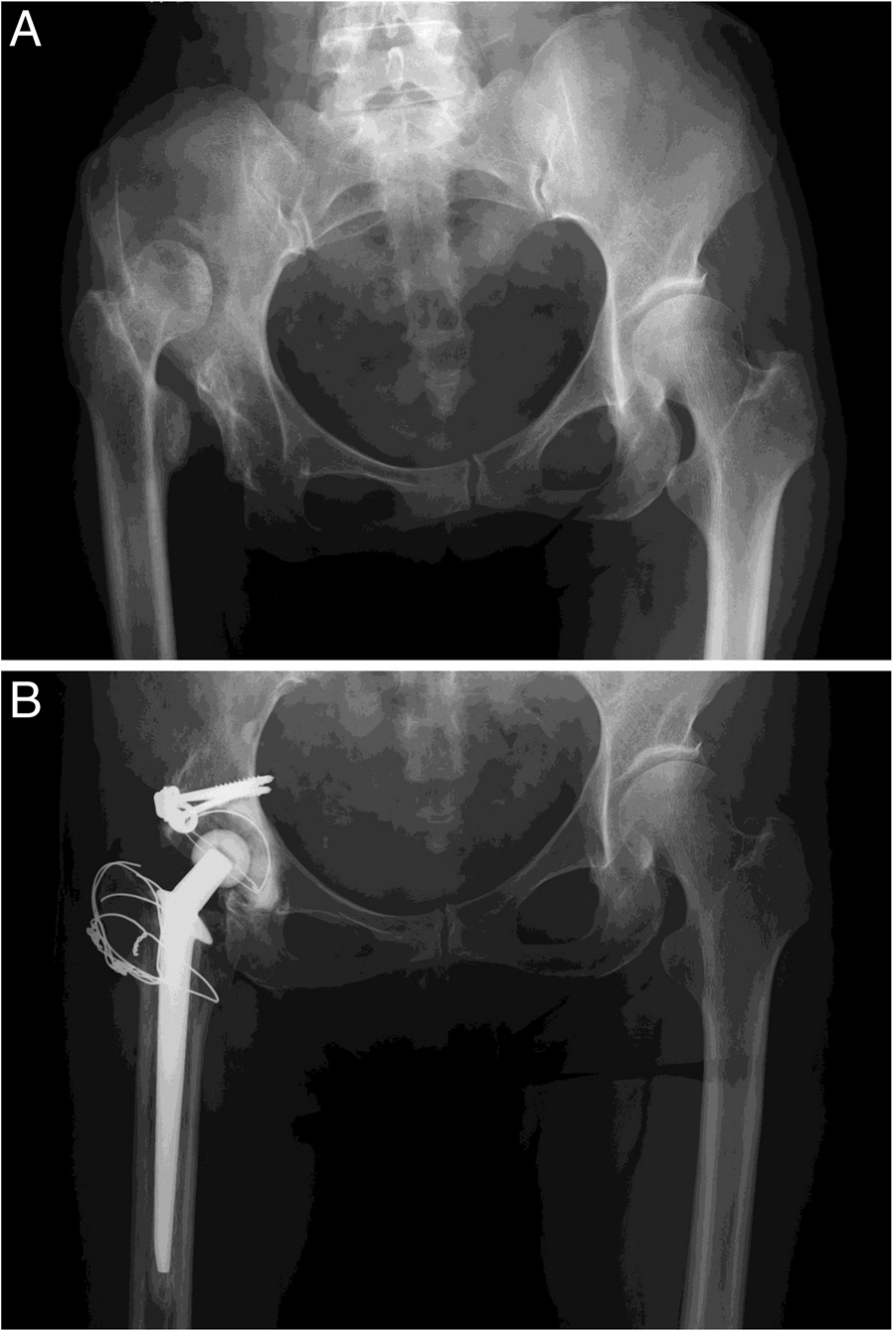
**Radiographs of a representative case. A**. An anteroposterior pelvic radiograph of a 57-year-old woman with Crowe IV complete dislocation of the right hip. **B**. A postoperative radiograph at 11 and a half years after a total hip arthroplasty with limb lengthening by 4.5 cm, showing no loosening or radiolucent line.

The mean percentage of bone graft for the acetabulum was 32% (19-47%) and mean limb lengthening 3.2 (1.0 to 5.1) cm. The leg length discrepancy was 2.7 (0–6.8) mm preoperatively and 0.6 (0–1.8) cm at final follow up. The mean correction of discrepancy does not exactly match the mean lengthening because in three patients the procedure was performed on both hips for Crowe IV and in 13 patients THA was performed on the contralateral hip for osteoarthritis (Crowe I to III) before final follow up. The rotational center of the hip was reconstructed inferiorly and medially after THA. The average horizontal distance of the postoperative center of the femoral head was 42.3 (30–61) mm preoperatively and 23.5 (19–34) mm postoperatively. The average height of the hip center was 62.5 (51–90) mm preoperatively and 21.7 (14–34) mm postoperatively. These results, according to Russotti and Harris [19], indicated that none of the hips had a high hip center, which is defined as more than 35 mm above the interteardrop line. Figure 2A and 2B show radiographs of a case in this series.

Complications were as follows. There were three complications involving the greater trochanter, which included two cases of greater trochanter avulsion resulting from femoral osteolysis in Zone 1 that was caused by debris from the Dall-Miles braided cables used for reattachment of the greater trochanter. There was one case of greater trochanter nonunion with a gap of less than 1 cm, but this did not manifest any symptoms. In this asymptomatic non-union case, monofilament titanium wires had been used for reattachment of the greater trochanter.

Two hips underwent revision surgery: one required isolated stem revision at 13 years after the initial procedure for the abovementioned avulsion of the greater trochanter that occurred subsequent to femoral osteolysis, and the other hip required revision surgery at 18 months after the initial procedure because of early collapse of the acetabular grafted bone. In the latter case, the femoral head used as a graft had been atrophic and possibly too weak, leading to early collapse. This case required a cup revision and reattempted bone grafting, which slightly changed the location of the rotation center and necessitated a stem revision to adjust the limb length. There were no cases of dislocation, infection, or nerve palsy. Among the 27 hips, 6 underwent limb lengthening of more than 4 cm during the THA. Among those 6 hips, 4 had the abovementioned complications including the two that required revision surgery.

Radiographic analysis demonstrated a partial radiolucent line on the acetabular cement-bone interface of approximately 1 mm in two hips (one hip in Zone I, the other in Zone III).

**Figure 3:**
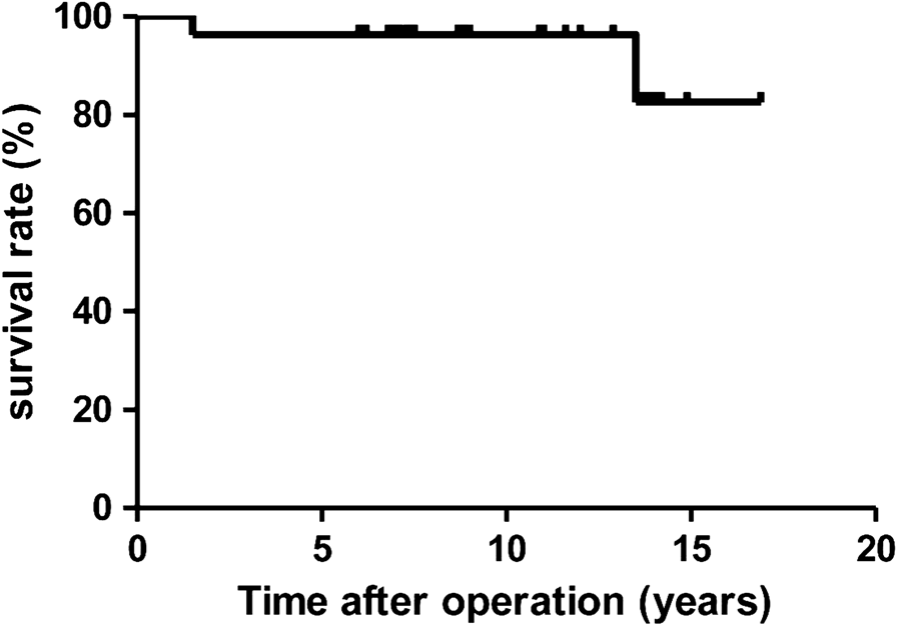
Survivorship with any revision surgery as the endpoint.

Survivorship analysis, with any revision as the end-point, gave a survival rate of 96.3% (95% confidence interval, 76.5 - 99.5%) at 10 years (Figure 3).

Dall-Miles cables were used in two hips, and these led to complications caused by metal debris from the cables. One hip showed a massive femoral osteolysis in the vicinity of the cable causing avulsion of the greater trochanter and necessitating revision surgery at 13 years after the first procedure. The other hip also had moderate femoral osteolysis leading to avulsion of the greater trochanter; this hip currently remains stable without revision surgery at 15 years of follow-up. In contrast, monofilament stainless wires were used in 24 hips, none of which developed osteolysis or non-union, although one hip suffered early collapse of the bone graft placed on the acetabulum, necessitating cup revision. Monofilament titanium wires were used in one hip, which had trochanteric non-union with a gap of less than 1 cm.

All except one of the acetabulars graft were considered to be united at from 6 to 18 months. One of the graft bones, as mentioned above, gradually collapsed within 18 months postoperatively and required revision surgery.

## Discussion

Most recent studies of THA for Crowe IV hips have included various types of shortening osteotomy, and these techniques have improved the outcome in such cases. However, there have been few reports on the long-term results of THA with shortening osteotomy [20-22]. Although these results seem encouraging, the long-term outcome at more than 10 years after THAs with shortening osteotomy remains unknown.

Regarding outcomes of THAs without shortening osteotomy for Crowe IV hips, Kerboull et al. reported a survival rate of 78% at 20 years with revision for any reason as the endpoint [12]. Numair et al. [11] reported higher rates of acetabular revision in completely dislocated hips, as compared with dysplastic hips. Chougle et al. reported that the cup survival rate in Crowe IV hips was 60.9% at 10 years and 15.6% at 20 years [23]. Compared with these reports, our results seem satisfactory indicating a survivorship of 96.3% at 10 years, with any revision as the endpoint.

The most serious risk associated with THA for Crowe group IV hip dislocation is nerve palsy. To avoid this risk, femoral shortening may be required in some cases. In this series, although the leg was lengthened by 3.2 cm on average, no hips developed nerve palsy. We do not favor muscle release or tenotomy to reduce the hip and always take great care to retain all of the periarticular muscles. The joint capsule, fibrous scar tissue, shelf, and osteophytes were completely removed in the present series. After total capsulectomy psoas muscle can be more extensible. Care was also taken not to pull the leg to reduce the femoral head into the cup, but instead to push the inserted stem head toward the cup center using a head pusher (Biomet, Warsaw, IN) with the hip and the knee both flexed at 30 degrees in order to avoid any excessive traction force on nerves. These approaches may be the reason for the absence of any nerve palsy in the present series.

Another reason to introduce femoral shortening osteotomy in our institute was to avoid problems arising from reattachment of the greater trochanter. The transtrochanteric approach provides wide exposure of the hip, thus facilitating accurate placement of the socket at the level of the true acetabulum in highly dislocated hips. This approach, however, may create problems resulting from reattachment of the greater trochanter when a large amount of limb lengthening is required. Without shortening osteotomy, more extensive resection of the femur to the level of the lesser trochanter is required for reduction in some cases. Extensive resection results in a small trochanteric bed, which may cause pseudoarthrosis of the greater trochanter. We believe that preservation of the trochanteric bed and careful reattachment are important for sound union of the greater trochanter.

Although some authors have reported that limb lengthening should be limited to 4 cm, or even 2 cm, Kerboull et al. [12] reported that limb lengthening to 7 cm was possible. In the present series, bringing the hip down to the level of the true acetabulum and limb lengthening of more than 4 cm were performed in six hips, none of which developed nerve palsy. These six hips developed four complications necessitating two revisions, whereas 21 the hips with limb lengthening of less than 4 cm needed no revision, and none developed osteolysis, acetabular loosening, or greater trochanter non-union. These results can be interpreted as indicating that limb lengthening by more than 4 cm undermines the long-term result even when the rotation center is successfully placed at the level of the true acetabulum without causing nerve palsy. However, among the four complications encountered, three involved problems with the greater trochanter, including two incidences of greater trochanter avulsion accompanied by debris from braided cables leading to massive femoral osteolysis, and one case of stable non-union causing no symptoms and requiring no treatment. These complications suggest that a smaller trochanteric bed resulting from extensive femoral neck resection and the use of braided cables for reattachment of the greater trochanter may be major factors that affect outcomes.

Limb lengthening of > 4 cm itself does not necessarily lead to problems with the greater trochanter. In our previously reported series of 17 hips that underwent THA via the transtrochanteric approach with subtrochanteric transverse osteotomy, the level of the greater trochanter was lowered by 7.6 cm on average (4.3 to 10.6 cm). Although in all 17 hips the greater trochanter was lowered by more than 4.3 cm, no trochanteric non-union or osteolysis occurred [9]. Taken together, these previous results and the present ones suggest that complications involving the greater trochanter result from not only the amount of limb lengthening itself but also the use of braided cables and a smaller trochanteric bed resulting from resection of the femoral neck.

The use of Dall-Miles braided cables to reattach the greater trochanter could have adversely affected the results. Whereas Dall-Miles braided cables were used in two hips, monofilament metal wires were used in the remaining cases. Among the two hips treated with braided cables, one needed stem revision at 13 years after the initial procedure due to massive femoral osteolysis involving the greater trochanter, and the other also showed femoral osteolysis leading to avulsion of the greater trochanter. While several modalities for cerclage fixation are in widespread use, the metal composition imposes potential complications, including third-body generation, and accelerated wear of the bearing surface. Several studies have documented poor outcomes of THAs that were performed with braided cables [24,25]. However, we cannot conclude that the use of braided cables had a significant impact on outcome in the present study because of the small number of cases involved. Some of the other major limitations of this study included the retrospective nature of data collection, and the relatively small sample population to measure the survival rate precisely, which resulted in the broad 95% confidence interval of 76.5 - 99.5% for 10-year survival.

## Conclusions

THA for Crowe IV hips without shortening osteotomy provided satisfactory results. However, three out of 27 hips developed complications that involved the greater trochanter. Especially, when braided cables were used to secure the greater trochanter, they led to osteolysis in the long term. Although no nerve palsy occurred in this series, care should always be taken for nerve tension when lengthening the limb.

## Competing interests

The authors declare that they have no competing interest.

## Authors’ contributions

TK carried out radiographic analyses and drafted the manuscript. CT performed the operations and revised the manuscript. HK helped to develop the design of the study and contributed to the revision of the manuscript. All authors read and approved the final manuscript.

## Authors’ information

Investigation was done in Kyoto City Hospital, but first author currently belongs to Kyoto University.

## Pre-publication history

The pre-publication history for this paper can be accessed here:

http://www.biomedcentral.com/1471-2474/15/72/prepub
